# The dilemma of recalling well-circumscribed masses in a screening population: A narrative literature review and exploration of Dutch screening practice

**DOI:** 10.1016/j.breast.2023.05.001

**Published:** 2023-05-03

**Authors:** Tanya D. Geertse, Daniëlle van der Waal, Willem Vreuls, Eric Tetteroo, Lucien E.M. Duijm, Ruud M. Pijnappel, Mireille J.M. Broeders

**Affiliations:** a Dutch Expert Centre for Screening (LRCB), Wijchenseweg 101, 6538 SW, Nijmegen, the Netherlands; b Canisius Wilhelmina Hospital, Department of Radiology Weg Door, Jonkerbos 100, 6532 SZ, Nijmegen, the Netherlands; c Amphia Hospital, Department of Radiology Molengracht 21, 4818 CK, Breda, the Netherlands; d University Medical Centre Utrecht, Utrecht UniversityDepartment of Radiology, Heidelberglaan 100, 3584 CX, Utrecht, the Netherlands; e Radboud University Medical CenterDepartment for Health Evidence Geert Grooteplein 21, 6525 EZ, Nijmegen, the Netherlands

**Keywords:** Breast cancer, Screening population, Well-circumscribed masses, Probably benign lesions, False positive screening outcomes

## Abstract

**Background:**

In Dutch breast cancer screening, solitary, new or growing well-circumscribed masses should be recalled for further assessment. This results in cancers detected but also in false positive recalls, especially at initial screening. The aim of this study was to determine characteristics of well-circumscribed masses at mammography and identify potential methods to improve the recall strategy.

**Methods:**

A systematic literature search was performed using PubMed. In addition, follow-up data were retrieved on all 8860 recalled women in a Dutch screening region from 2014 to 2019.

**Results:**

Based on 15 articles identified in the literature search, we found that probably benign well-circumscribed masses that were kept under surveillance had a positive predictive value (PPV) of 0–2%. New or enlarging solitary well-circumscribed masses had a PPV of 10–12%. In general the detected carcinomas had a favorable prognosis. In our exploration of screening practice, 25% of recalls (2133/8860) were triggered by a well-circumscribed mass. Those recalls had a PPV of 2.0% for initial and 10.6% for subsequent screening. Most detected carcinomas had a favorable prognosis as well.

**Conclusion:**

To recognize malignancies presenting as well-circumscribed masses, identifying solitary, new or growing lesions is key. This information is missing at initial screening since prior examinations are not available, leading to a low PPV. Access to prior clinical examinations may therefore improve this PPV. In addition, given the generally favorable prognosis of screen-detected malignant well-circumscribed masses, one may opt to recall these lesions at subsequent screening, if grown, rather than at initial screening.

## Highlights

• Well-circumscribed masses are very common in breast cancer screening.
• Most well-circumscribed masses have a low positive predictive value for malignancy.
• Identifying solitary, new or growing lesions is key to recognizing malignancies.
• Comparison to prior clinical examinations may improve positive predictive value.
• Recall at subsequent rather than initial screening, if grown, could be considered.

## 1 Introduction

Mammographic breast cancer screening in combination with state-of-the art treatment is still the most effective strategy for a substantial reduction in mortality from this disease. Detection at an earlier stage results in less invasive treatment and improved survival [1,2]. If an abnormality suspicious for cancer is seen on the screening mammogram, the woman is recalled for further assessment. In the Netherlands, the recall rate continuously increased over the years [3,4]. This resulted in an increase in the cancer detection rate, but also in a disproportionate increase in false positive recalls, especially at initial (or first) screening examinations (see figure in Appendix A) [4]. False positive recalls cause anxiety, a lower re-attendance rate, and additional costs [[5], [6], [7]].

The result of a screening examination is classified using the Breast Imaging Reporting And Data System (BI-RADS) lexicon [8]. BI-RADS category 0, adapted for use in a screening setting, represents an abnormality with a low suspicion for cancer. It is assigned to recalls related to well-circumscribed masses, architectural distortions seen in one direction, and asymmetries [8]. Half of all recalls in the Dutch screening program are classified as BI-RADS 0 (2019: 12.6 per 1000 of 23.9 per 1000 recalls [52.7%]) [4]. For these BI-RADS 0 recalls, the positive predictive value of recall (PPV) was found to be 10%. Thus, in 90% of these women there was no cancer diagnosed after further assessment. Of these women with a false positive recall, 15% underwent a diagnostic biopsy [4]. Although the PPV of the separate radiological features classified as BI-RADS 0 is unknown, well-circumscribed masses are very common in a screening population (approximately 8% of all screening mammograms [9]). Well-circumscribed masses with a typically benign appearance, such as typical intramammary nodes, hamartomas, and oily cysts, are easily recognized by screening radiologists and do not have to be recalled for further assessment. But the vast majority of the well-circumscribed masses are not typically benign and fall into the “probably benign” category. In this category, it is harder for screening radiologists to decide whether further assessment is necessary, knowing that the PPV is very low (<2%) [10,11].

The Dutch recall strategy indicates that solitary, new, or growing well-circumscribed masses should be recalled (BI-RADS 0) for further assessment. To determine whether the number of false positive recalls can be reduced by improving the recall strategy, we need to better understand the clinical relevance of well-circumscribed masses. Although several studies have been published on this topic, to our knowledge, no review of the literature has been performed to combine all available evidence.

The aim of this study is to investigate the characteristics of malignancies presenting as well-circumscribed masses on mammography, in order to identify the potential room for improvement in the recall strategy, particularly for initial screening. We report the results of a narrative literature review and the follow-up results of all screening examinations assigned a BI-RADS 0 in a Dutch screening region in the period 2014 to 2019.

## 2 Materials and methods

### 2.1 Literature review

A systematic search was performed in April 2021, using PubMed (Appendix B), and updated in April 2022. Key search terms included: “mammography”, “well-circumscribed mass”, and variations of these terms. There were no restrictions regarding the type of journal or publication date. Articles written in a language other than English or Dutch were excluded. Titles and abstracts were screened to determine relevance. We reviewed the references of all relevant articles (snowballing) for additional ones. Articles were included if they met the following inclusion criteria: (1) the study population consisted of women (with or without symptoms) undergoing periodic mammography examination (for screening or follow-up of probably benign lesions); and (2) the outcome of assessment of the well-circumscribed masses presenting on the mammography examinations was reported (at least cancer or no cancer). We excluded studies if they focused on women with a mutation in one of the breast cancer susceptibility genes.

### 2.2 Exploration of screening practice

The screening organization in the south of the Netherlands provided data on all women who participated in the Eindhoven region between January 1, 2014, and January 1, 2019, and who were recalled based on a lesion on their screening mammogram. The anonymized data included information on the screening outcome and the clinical assessment (radiology, pathology, and surgical procedures performed at a hospital after the recall). By participating in screening, women consent to their data being made available for evaluation purposes and research, unless they choose to opt out explicitly. We did not receive any data of women who objected to the use of their data. This study was performed under the national permit for breast cancer screening issued by the Ministry of Health, Welfare and Sports and did not require additional approval by a local institutional review board.

Details of the Dutch national breast cancer screening program have been described previously [[12], [13], [14]]. In short, participating women get a two-view full-field digital mammogram (Lorad Selenia, Hologic). All mammograms are performed by a radiographer specialized in mammography. Each mammogram is read by two certified screening radiologists independently. Only for subsequent screening examinations, prior examinations are available for comparison. Mammograms are classified according to the BI-RADS lexicon [8]. BI-RADS 1 or 2 implies no recall, whereas women with a BI-RADS 0, 4, or 5 are recalled for clinical assessment. The screening program does not allow a BI-RADS 3 since no short-term follow-up is available in the screening setting.

Only women with a BI-RADS 0 recall based on a well-circumscribed mass, according to the recall letter, were included in the analyses. The outcome of the clinical assessment of these women was evaluated. Multiple foci of cancer in one breast were counted as one cancera. Due to the exploratory nature of this study, only descriptive statistics are presented here.

## 3 Results

### 3.1 Literature review

**Fig. 1:**
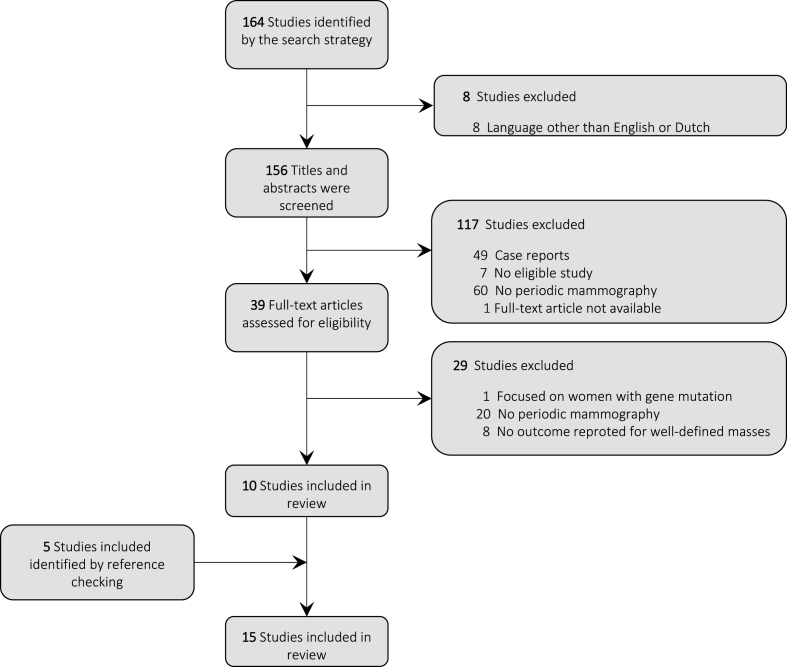
Flowchart of the literature search.

The search identified 164 articles (Fig. 1). After title and abstract screening and reference checking, a total of 15 articles was included in the review. The update in March 2023 did not yield any additional articles.

Table 1 shows the outcomes of the well-circumscribed masses described in the included articles. The study objectives of the selected articles were very heterogeneous. The number of cases related to well-circumscribed masses varied greatly, ranging from 24 to 1440. The number of cancers detected related to these well-circumscribed masses ranged from 0 to 91. The study populations consisted of women undergoing periodic mammography examination in the context of breast cancer screening (n = 12 studies) [9,17,[19], [20], [21], [22], [23], [24], [25], [26], [27], [28]] or surveillance for a probably benign lesion (n = 3 studies) [15,16,18].

**Table 1:** Characteristics of well-circumscribed masses of the selected articles

Reference	Study objective	Country	Population	Age	Sample size	Cases well-circumscribed masses	Number of breast cancers	Proportion of breast cancers	Type of breast cancer	Histological grade	Stage	Lymph node status	Receptor status
**Sickles, 1991** [15]	Establish the validity of managing probably benign lesions with periodic mammographic surveillance	USA	Women (asymptomatic or symptomatic) who underwent periodic mammographic surveillance for a probably benign lesion (during a 8.5-year period)	Range 28-96, Median 51	3,184 women	842 589 (one) 253 (multiple)	13 12 1	1.5%^a^ 2.0% 0.4%	Only reported for all probably benign lesions combined	NR	Only reported for all probably benign lesions together	One demonstrated axillar lymph node metastasis	NR
**Datoc, 1991** [16]	Compare the efficacy of single-view and two-view examinations for the follow-up of mammographic findings associated with low suspicion for malignancy	USA	Women who underwent periodic mammographic surveillance for a probably benign lesion (6 months follow-up)	Range 26-81, Mean 53.7	498 women with a total of 666 mammographic abnormalities	314 masses of 666 abnormalities	0	0%	NA	NA	NA	NA	NA
**Opie,** **1993** [17]	Determine the yield of carcinoma in patients with a nonpalpable mammographic abnormality and identify which mammographic criteria will most likely yield a positive biopsy	USA	Women participating in screening who had a nonpalpable abnormality detected and biopsied	Range 24-86	295 women who underwent 332 biopsies	47 masses	5	10.6%	Only reported for all lesions combined	NR	Only reported for all lesions together	NR	NR
**Sickles, 1994** [18]	Determine whether lesion size and patient age should prompt immediate biopsy of nonpalpable, circumscribed, noncalcified solid breast masses	USA	Women (asymptomatic or symptomatic) who underwent periodic mammographic surveillance for a probably benign lesion (during a 12.3-year^b^ period)	Range 28-94, Median 50	58,415 mammograms (a woman can have more than one mammogram)	1,403	19	1.4%	16 IBC-NST (84%) 3 DCIS (16%)	NR	3 stage 0 (16%) 14 stage I (74%) 2 stage II (11%)	One demonstrated axillar lymph node metastasis	NR
**Burrell, 1996** [19]	Identify factors which may improve sensitivity and specificity of mammographic interpretation	UK	Women (asymptomatic or symptomatic) participating in screening who had a nonpalpable abnormality detected and biopsied	Range 30-75, Mean 55	416 women who underwent 425 biopsies (303 asymptomatic + 122 symptomatic)	24	Asymptomatic 0 Symptomatic 1	Asymptomatic 0% Symptomatic 4.2%	1 Intracystic carcinoma (100%)	Only reported for all lesions together	Only reported for all lesions together	NR	NR
**Hussain, 1999** [20]	Assess the nature of new densities and microcalcifications in the second round of breast screening	UK	Women participating in screening (2nd round), with abnormalities not present in 1^st^ round	Range 50-64	311 lesions identified in 302 women	53	2	3.8%	Only reported for all lesions together	Only reported for all lesions together	NR	Only reported for all lesions together	NR
**Leung, 2000** [21]	Assess the need for recalling women with multiple masses	USA	Women (asymptomatic) participating in screening with multiple bilateral masses	NR	84,615 examinations of 40,419 women	1440 examinations with multiple masses among 907 women	4	0.4%	3 IBC-NST (75%) 1 Mucinous (25%)	2 Grade 1 (50%) 1 Grade 2 (25%) 1 Grade 3 (25%)	3 stage I (75%) 1 stage IIa (25%)	3 negative (75%) 1 positive (25%)	NR
**Dhillon, 2006** [22]	Describe the imaging features of 34 screen-detected mucinous carcinomas	Australia	Women (asymptomatic) participating in screening and with a screen-detected mucinous carcinoma	Range 48-82 Mean 65	214,507 women 2745 invasive cancers 45 mucinous cancers (11 mucinous cancers were excluded, 34 were described)	30	30	NA	Mucinous	30 Grade 1 or 2 (100%)	NR	30 negative (100%)	NR
**Farshid, 2008** [9]	Establish the reliability of FNAB as a first line diagnostic modality for assessment of category 3 screen-detected mass lesions	Australia	Women (asymptomatic) participating in screening and with a category 3B^c^ solid circumscribed mass	Range 50-69	1,183 lesions (538 initial screening, 645 subsequent screening)	1,183	91^d^	7.7% (3% initial screening 13% subsequent screening)^e^	49 IBC-NST (54%) 13 Mucinous (14%) 2 Tubular (2%) 1 Medullary (1%) 1 Inv. Lobular (1%) 25 DCIS (27%)	Invasive^f^: 49.2% Grade 1 33.3% Grade 2 17.5% Grade 3 DCIS: 16.0% high grade	NR	NR	NR
**Bonetti, 2008** [23]	Confirm that FNAC is a reliable first diagnostic tool for the assessment of breast lesions	Italy	Women participating in screening and with a category 3B^5^ solid circumscribed mass	NR	388 lesions	388zzzz	18^g^	4.6%	9 IBC-NST (50%) 5 Mucinous (28%) 1 Medullary (6%) 1 Inv. Lobular (6%) 1 Inv. Papillary (6%) 1 DCIS (6%)	NR^h^	NR	NR	NR
**Bandan, 2013** [24]	Evaluate BI-RADS as a predictive factor for suspicion of malignancy in breast lesions by correlating radiological findings with histological results in a breast cancer reference hospital	Brazil	Women (asymptomatic or symptomatic) participating in screening and recalled for FNAB, core biopsy or vacuum-assisted core biopsy	Range 16-84, Mean 49	580 women (recalls) 276 BI-RADS 3^i^ 230 BI-RADS 4 74 BI-RADS 5	248	2	0.80%	Only reported for all lesions together	NR	NR	NR	NR
**Timmers, 2013** [25]	Develop a prediction model for breast cancer based on common mammographic findings on screening mammograms, aiming to reduce reader variability in assigning BI-RADS	Netherlands	Women participating in subsequent screening who were recalled	Range 53-75 Mean 62	352 women (recalls) 120 BI-RADS 0 198 BI-RADS 4 34 BI-RADS 5	60	6	10%	NR	NR	NR	NR	NR
**McDonald, 2017** [26]	Evaluate BI-RADS 3 assessment after recall from screening, before and after implementation of DBT Cohort 1: FFDM Cohort 2: DBT +FFDM	USA	Women (asymptomatic) participating in screening (without symptoms or physical examination findings and no prior history of breast cancer) and recalled	Range <40 - >69 App. 85% 40-69 Cohort 1: Mean 54.2 Cohort 2: Mean 53.8	Cohort 1: 184 BI-RADS 3 Cohort 2: 227 BI-RADS 3	Cohort 1: 41 Cohort 2: 61	Cohort 1: 1 Cohort 2: 0	Cohort 1: 2.4% Cohort 2: 0%	1 IBC-NST (100%)	NR	NR	NR	NR
**Nakashima, 2017** [27]	Compare the visibility of circumscribed masses on DBT images and 2D mammograms and determine the usefulness of DBT for differentiation between benign and malignant circumscribed masses	Japan	Women participating in screening who were recalled	Malignant lesions: Mean 61 Benign lesions: Mean 53	1395 women (recalls)	115	19	17%	10 IBC-NST (53%) 2 DCIS (11%) 3 Papillary (16%) 2 Metaplastic (11%) 1 Mucinous (5%) 1 Phyllodes (5%)	NR	NR	NR	NR
**Stepanek, 2019** [28]	Compare the utilization of BI-RADS 3 assessment after recall from screening before and after implementation of DBT Cohort 1: FFDM Cohort 2: DBT +FFDM	USA	Women participating in screening who were recalled	Range <40 - >69 App. 85% 40-69	BI-RADS 3 Cohort 1: 388 women (463 lesions) Cohort 2: 220 women(254 lesions)	Cohort 1: 83 Cohort 2: 47	Cohort 1: 2 Cohort 2: 1	Cohort 1: 2.4% Cohort 2: 2.1%	1 DCSI (50%) 1 IBC-NST (50%) 1 Papillary (100%)	NR	1 Stage 0 (50%) 1 Stage IA (50%) 1 Stage 0 (100%)	Negative Negative Negative	ER+PR+ ER-PR-HER2- ER+PR+

**Abbreviations:** NR = not reported; NA = not available; IBC-NST = invasive breast carcinoma of no special type; DCIS = ductal carcinoma in situ; LCIS = lobular carcinoma in situ; Inv.= invasive; FNAB = fine needle aspiration biopsy; FNAC = fine needle aspiration cytology; BI-RADS = Breast Imaging Reporting And Data System; FFDM = full field digital mammography; DBT = digital breast tomosynthesis; ER=estrogen receptor; PR=progesterone receptor; HER2=human epidermal growth factor receptor 2

a PPV=11.5%, when a lesion changes on mammography or becomes palpable. No cancers were found among biopsy without mammographic change.

b This 12.3-year period includes the 8.5-year period of the study of 1991.

c According to the Tabar 5-tier grading scheme.

d Not included here: 1 LCIS, 1 Leiomyosarcoma, 3 Lymphoma, 1 Metastasis.

e In 2011 Farshid et al. published an extension of this study from 2008 [26], in which these percentages are mentioned.

f Numbers are not reported.

g Not included here: 1 LCIS

h It was only reported that no highly aggressive tumors were observed in the series.

i Nodules with circumscribed margins, clustered punctiform microcalcifications, and focal asymmetry without associated findings.

Four studies explicitly mentioned that all participants were asymptomatic [9,21,22,26], four studies described in their method section that women with symptoms were included as well [15,18,19,24], and the other seven studies did not report information about symptoms [16,17,20,23,25,27,28].

In all studies, well-circumscribed masses generally had a benign outcome. The highest reported PPV was 17% [27]. This high PPV can probably be explained by the fact that only mammographically detected well-circumscribed masses that could also be detected on ultrasound or MRI were examined in this study. In the other studies, the PPV varied from 0% to 10.6%. To gain more insight into the clinical relevance of probably benign lesions, Sickles distinguished solitary from multiple well-circumscribed masses [15]. The PPV was 2.0% for solitary well-circumscribed masses and 0.4% for multiple masses. Later, Leung conducted a study together with Sickles to assess the need to recall women with multiple masses [21]. For this study they included a different population than in the previous study by Sickles [15]. They again found a PPV of 0.4%. Sickles [15] also described that the PPV increased to 11.5% when a well-circumscribed mass changed over time or became palpable. Opie et al. [17] reported that 10.6% of solid, enlarging well-circumscribed masses were malignant. Timmers et al. [25] reported similar findings: a PPV of 10% for well-circumscribed masses. This study was performed within the Dutch national breast cancer screening program and included only subsequent screening examinations. Most of the well-circumscribed masses in this study were therefore solitary, new or growing. In a study by Burrell et al. [19] the population was stratified into asymptomatic and symptomatic women. For the well-circumscribed masses, the PPV was 0% in asymptomatic women and 4.2% in symptomatic women. It should be noted that were only 24 mammography examinations showing a well-circumscribed mass.

Only eight studies [9,18,19,[21], [22], [23],^27^,^28^] reported the type of cancer related to the well-circumscribed masses. The study by Dhillon et al. [22] only included mucinous carcinomas. Due to the low prevalence, the number of cancers diagnosed among well-circumscribed masses in the smaller studies was very low. The larger studies [9,18,23,27] reported 19, 91, 18, and 19 breast cancers, respectively, of which at least 50% were invasive breast carcinoma of no special type (IBC-NST) (16/19 [85%], 49/91 [54%], 9/18 [50%], and 10/19 [53%], respectively). Besides IBC-NST, several of these four larger studies also mentioned mucinous (0/19 [0%], 13/91 [14%], 5/18 [28%], and 1/19 [5%], respectively), papillary (0/19 [0%], 0/91 [0%], 1/18 [6%], and 3/19 [16%], respectively) and ductal carcinoma in situ (DCIS) (3/19 [16%], 25/91 [27%], 1/18 [6%], and 2/19 [11%], respectively) as cancer type. Farshid et al. [9] found that many of the DCIS cases had a papillary component.

Only two of the larger studies [9,18] reported prognostic factors: histological grade, tumor stage, lymph node status, or receptor status. In the study by Sickles [18], 88% (14/16) of invasive cancers were stage I, and an axillary lymph node metastasis was found in only one case (6%). In the study by Farshid et al. [9] grade 1 tumors were most common, accounting for 49.2% (31/63) of the cases. Furthermore, 33.3% (21/63) were grade 2, and only 17.5% (11/63) of the cancers were grade 3. Grade was not specified for three cases. Of the 25 DCIS cases that presented as a well-circumscribed mass, only four were of high grade. The 30 mucinous carcinomas described in the study by Dhillon et al. [22] were all grade 1 or 2 and all had a negative lymph node status.

### 3.2 Exploration of screening practice

**Fig. 2:**
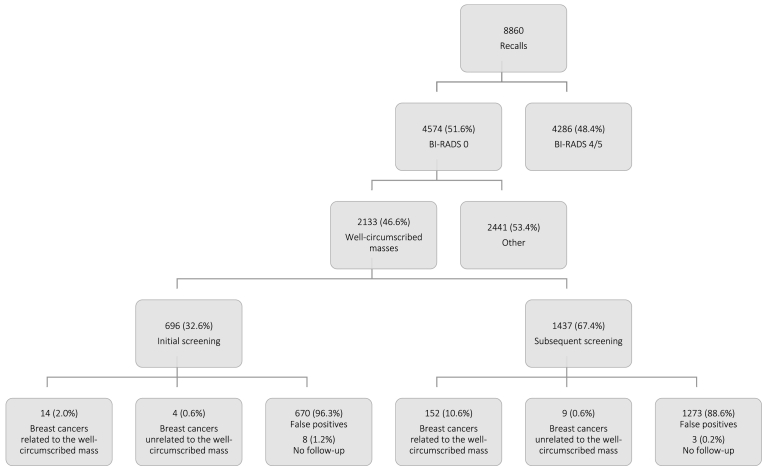
Screening mammography results of women recalled with BI-RADS 0 based on a well-circumscribed mass in a Dutch screening region in het period 2014–2019.

Of the 8860 recalled women in the database we used to explore screening outcomes, 4574 (51.6%) were recalled for a BI-RADS 0 lesion (see Fig. 2). Of these BI-RADS 0 lesions, 2133 (46.6%) presented as a well-circumscribed mass on the screening mammogram. A total of 179 (8.0%) women were diagnosed with breast cancer after recall for a well-circumscribed mass, 18 cancers at initial screening (PPV: 18/696 [2.6%]) and 161 cancers at subsequent screening (PPV: 161/1437 [11.2%]). In 13 of these 179 women, the cancer appeared to be unrelated to the well-circumscribed mass. Of these 13 women, eight women had a bilateral recall, with breast cancer diagnosed in the contralateral breast from where the well-circumscribed mass was detected. In the remaining five women, a malignancy was discovered elsewhere in the recalled breast (an incidental finding). These 13 women were excluded from our analyses. At subsequent screening, 152 breast cancers diagnoses were related to a well-circumscribed mass (PPV: 152/1437 [10.6%]). At initial screening, breast cancer related to a well-circumscribed mass was diagnosed in 14 of 696 screening examinations (PPV: 2.0%).

Table 2 shows the details of the 166 breast cancers related to a well-circumscribed mass. Most cancers were IBC-NST (initial screening: 10 of 14 cancers [71.4%]; subsequent screening: 107 of 152 cancers [70.1%]). We further observed invasive lobular carcinomas, but only in subsequent screening examinations (initial screening: 0 of 14 cancers [0%]; subsequent screening: 17 of 152 cancers [11.2%]). Of the rare subtypes of invasive cancers, mucinous (initial screening: 0 of 14 cancers [0%]; subsequent screening: 5 of 152 cancers [3.3%]) and tubular carcinomas (initial screening: 2 of 14 cancers [14.3%]; subsequent screening: 7 of 152 cancers [4.6%]) were most frequently encountered. DCIS was found in 10 women (initial screening: 2 of 14 women [14.3%]; subsequent screening: 8 of 152 women [5.3%]), 9 of these 10 women (90%) had a well-circumscribed mass such as fibroadenoma (n = 2) or papillary lesion (n = 7) with DCIS as an additional finding on biopsy.

**Table 2:** Tumor characteristics of 166 screen-detected cancers presenting as a well-circumscribed mass at screening mammography.

	Initial screening	Subsequent screening
Type of carcinomas	**n** = **14**	**n** = **152**
DCIS	2 (14.3%)	8 (5.3%)
IBC-NST	10 (71.4%)	107 (70.1%)
ILC	0 (0%)	17 (11.2%)
mixed IBC- NST/ILC	0 (0%)	5 (3.3%)
Inv tubular	2 (14.3%)	7 (4.6%)
Inv papillary	0 (0%)	1 (0.7%)
Inv mucinous	0 (0%)	5 (3.3%)
Inv mucinous/papillary	0 (0%)	1 (0.7%)
Inv neuroendocrine	0 (0%)	1 (0.7%)
Tumor size	**n** = **14**	**n** = **152**
Tis	2 (14.3%)	8 (5.3%)
T1A	2 (14.3%)	19 (12.5%)
T1B	7 (50.0%)	64 (42.1%)
T1C	2 (14.3%)	47 (30.9%)
T2	0 (0%)	11 (7.2%)
T3+	1 (7.1%)	3 (2.0%)
Grading		
DCIS	**n** = **2**	**n** = **8**
Low	2 (100%)	4 (50.0%)
Intermediate	0 (0%)	3 (37.5%)
High	0 (0%)	1 (12.5%)
Bloom&Richardson	**n** = **12**	**n** = **144**
I	7 (58.3%)	70 (48.6%)
II	5 (41.7%)	59 (41.0%)
III	0 (0%)	15 (10.4%)
Receptor status	**n** = **12**	**n** = **144**
ER+, PR+/−, HER2	11 (91.7%)	128 (88.9%)
ER+/−, PR+/−, HER2+	1 (8.3%)	9 (6.3%)
Triple negative	0 (0%)	7 (4.9%)
Lymph node status	**n** = **12**	**n** = **144**
Negative	8 (66.7%)	115 (79.9%)
Positive	3 (25.0%)	21 (14.6%)
Nx	1 (8.3%)	8 (5.6%)

**Abbreviations:** DCIS = ductal carcinoma in situ; IBC-NST = invasive breast carcinoma of no special type; ILC = invasive lobular carcinoma; Inv = invasive; ER = estrogen receptor; PR = progesterone receptor; HER2 = human epidermal growth factor receptor 2; Nx = cannot be measured.

The malignant well-circumscribed masses generally comprised cancers with a favorable prognosis. Most cancers were grade I or II (initial screening: 12 of 12 cancers [100%]; subsequent screening: 129 of 144 cancers [89.6%]) and had a negative lymph node status (initial screening: 8 of 12 cancers [66.7%]; subsequent screening: 115 of 144 cancers [79.9%]). The hormone receptor status of the invasive cancers was predominantly ER+, PR + or -, and HER2- (initial screening: 11 of 12 cancers [92%]; subsequent screening: 128 of 144 cancers [88.9%]). HER2+ breast cancer was found in 1 of 12 women [8.3%] at initial screening and in 9 of 144 women [6.3%] at subsequent screening. Triple negative breast cancers were rare (initial screening: 0 of 12 women [0%]; subsequent screening: 7 of 144 women [4.9%]).

## 4 Discussion

The narrative literature review showed that probably benign well-circumscribed masses at mammography had a PPV of 0–2%. When limited to new or growing well-circumscribed masses, the PPV increased to 10–12%. In general, the cancers detected had a favorable prognosis. Our exploratory study showed that almost 25% of all recalls were triggered by a well-circumscribed mass on the screening mammogram. We found a PPV of 2.0% for initial screening examinations, 10.6% for subsequent screening examinations, and 8.0% for all screening examinations combined. Thus, the majority of well-circumscribed masses were benign, especially for initial screening examinations (98.0%). In addition, and in line with the literature review, most cancers detected had a favorable prognosis.

The Dutch study by Timmers et al. [25] reported a PPV of 10% for subsequent screening examinations. Of all other studies included in our literature review, the recall strategy in the study by Farshid et al. [9], performed within the Australian breast cancer screening program, most closely resembles the recall strategy of the Dutch breast cancer screening program. The authors reported an overall PPV of 8% for well-circumscribed masses. In an extension of this study, Farshid et al. [29] found that the PPV was 3% for initial screening examinations and 13% for subsequent screening examinations, which is quite similar to results from Dutch screening practice. Unlike the recall policy in the Netherlands, in the USA all probably benign well-circumscribed masses are recalled and assigned a BI-RADS 3, for which short-term surveillance is recommended. A PPV of 0–2% [15,16,18,26,28] has been reported for this setting. During surveillance, a morphological changes or an increase in size is an indication for needle biopsy, resulting in a PPV of biopsy of 10–12% [15,17].

The difference in PPV between all probably benign well-circumscribed masses (0–2%) and those that are new or enlarging (10–12%) may at least partly explain the distinct difference between the PPV at initial (2.0%) versus subsequent (10.6%) screening examinations. The radiologists have no prior examinations to compare with during reading of initial screening examinations, resulting in the recall of more probably benign well-circumscribed masses. For subsequent screening examinations, the radiologists have prior examinations to compare with, which makes it possible to only recall new or enlarging well-circumscribed masses. This is true for the majority of the Dutch breast cancer screening population, because in the Netherlands the re-attendance rate is 91% [4].

The low PPV at initial screening examinations (2.0%) suggests that the balance between screen-detected cancers and false positive recalls is unfavorable. This balance could potentially be improved if a prior mammogram is available for comparison. Several studies have shown that, in breast cancer screening, the availability of prior mammograms for comparison reduces the false positive rate [[30], [31], [32], [33], [34]]. To our knowledge, no previous study has focused on initial screening. The extent to which prior mammograms could be made available at initial screening depends on how screening is organized. Most likely, women will have to give their consent for using their prior clinical mammograms for comparison. This can be facilitated by making women aware of the importance of providing their prior mammograms. A survey study by Horsley et al. reports that, even in a group of women who routinely underwent screening mammography, most women did not think that prior mammograms are important to decrease false positive recalls [35]. It is known that in the Netherlands a large proportion of women have had a mammogram in a clinical setting before reaching the screening starting age. Data from the Netherlands Institute for Health Services Research (NIVEL) show that yearly an estimated 1 in 50 women over the age of 25 have an appointment in the hospital because of fear of having breast cancer or breast problems [36,37]. In the Netherlands further assessment of recalled participants is performed in a hospital and is not part of the screening program. Privacy legislation can therefore be an obstacle in retrieving both medical history and/or clinical mammograms for comparison in screening.

In general, the pathological characteristics of cancers found in an asymptomatic screening population differ from symptomatic and interval cancers [38,39]. The poorer prognosis for interval cancers seem to be associated with their biological differences and more rapid tumor growth. The latter means that the preclinical detectable phase of high-grade carcinomas is often too short to be detected during screening. As a consequence, in screening in particular low-grade, slower growing carcinomas are detected, which could explain the mostly favorable prognosis of the screen-detected, malignant, well-circumscribed masses in our study. Given this generally favorable prognosis and the high re-attendance rate of 91% [4], it might be possible to wait and recall these lesions at subsequent screening, if grown, rather than recalling them at initial screening.

For the few rapidly growing and more aggressive carcinomas that present as a well-circumscribed mass at the time of screening, we need to find a mammographic feature that is able to identify these cancers and avoid a delay in detection. It is quite conceivable, that in the coming years new artificial intelligence algorithms will be developed which can help radiologists to identify these cancers.

Our study has several strengths and limitations. An important strength of this study is that it combines a literature review with an exploration of actual screening practice based on a large sample size. An important limitation of this study is that data on well-circumscribed masses was scarce and did not allow us to draw strong conclusions based on the few, mostly small, studies, identified by the literature search. In addition, for the exploratory study, the presence of a well-circumscribed mass was only based on the description in the recall letters, drafted by the screening radiologists, and could not be based on radiological review of the mammograms.

## 5 Conclusions

To recognize malignancies presenting as well-circumscribed masses, identifying solitary, new or growing lesions is key. This information is missing at initial screening since prior examinations are not available, resulting in a low PPV. Access to prior clinical examinations may therefore improve this PPV. In addition, given the generally favorable prognosis of screen-detected, malignant, well-circumscribed masses, one may opt to recall these lesions at subsequent screening, if grown, rather than at initial screening.

## Declaration of competing interest

The authors of this manuscript certify that they have no affiliations with or involvement in any organization or entity with any financial interest (such as honoraria; educational grants; participation in speakers’ bureaus; membership, employment, consultancies, stock ownership, or other equity interest; and expert testimony or patent-licensing arrangements), or non-financial interest (such as personal or professional relationships, affiliations, knowledge or beliefs) in the subject matter or materials discussed in this manuscript.

This research did not receive any specific grant from funding agencies in the public, commercial, or not-for-profit sectors.

This study was performed under the national permit for breast cancer screening issued by the Ministry of Health, Welfare and Sports and did not require additional approval by a local institutional review board.

## Figures and Tables

**Figure:**
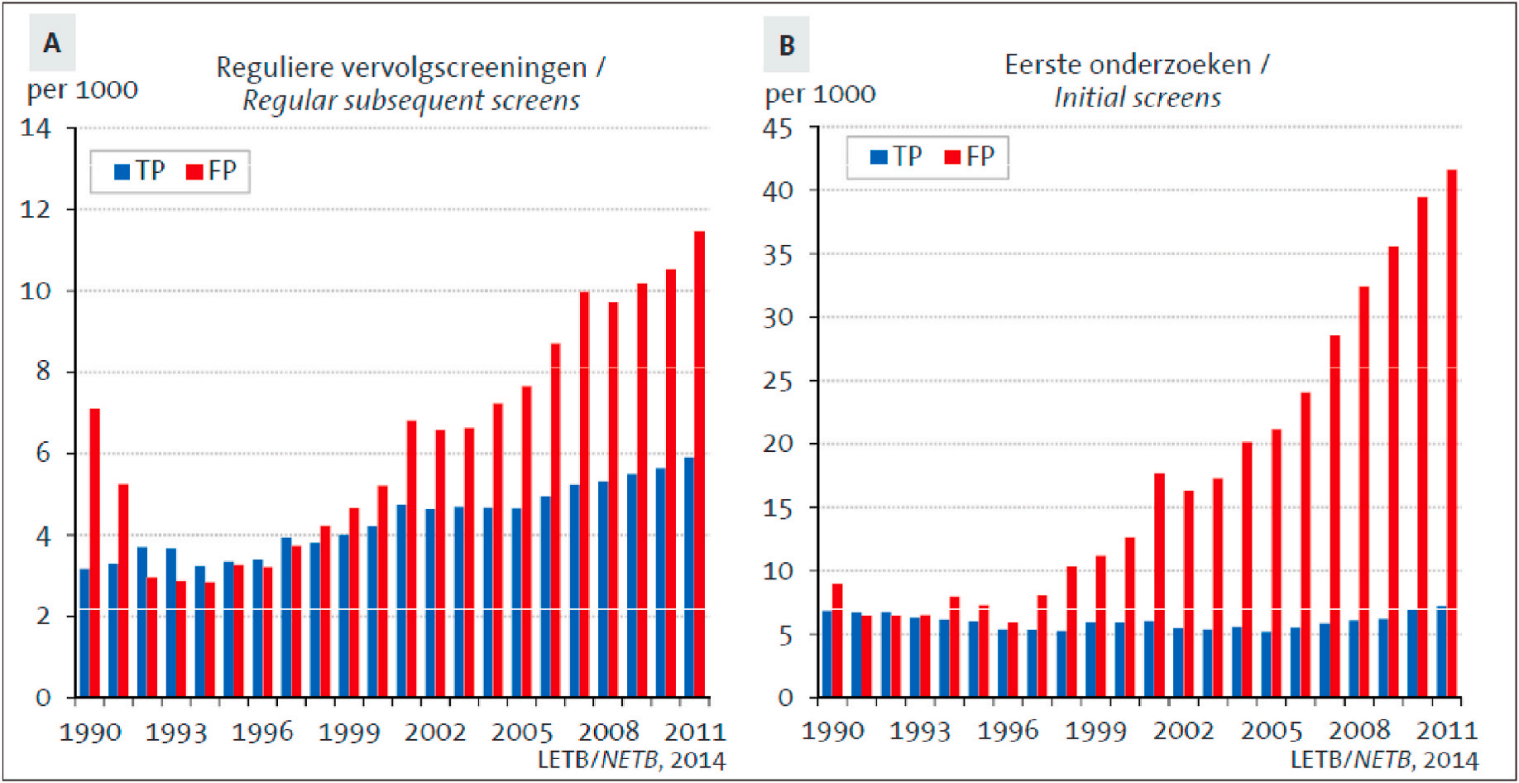
True-positive (TP) and false-positive (FP) screen results per 1000 women screened for A: regular subsequent, and B: initial screens, 1990-2011

## Appendix A True-positive (TP) and false-positive (FP) screen results per 1000 women screened from the Dutch breast cancer screening 1990–2011

Source: Fig. 3.9 from the report “National evaluation of breast cancer screening in the Netherlands 1990–2011/2012: Thirteenth evaluation report”, published by the National Evaluation Team for Breast cancer screening in April 2014.

## Appendix B Search Strategy

We performed the following search strategies in PubMed:

Component 1 = breast cancer.

Component 2 = mammography.

Component 3 = well-defined mass.

(1) (Breast Neoplasm*[tiab] OR Breast Tumo*[tiab] OR Breast Cancer*[tiab] OR Mammary Cancer*[tiab] OR Malignant Neoplasm of Breast [tiab] OR Breast Malignant Neoplasm*[tiab] OR Malignant Tumor of Breast [tiab] OR Breast Malignant Tumo*[tiab] OR Cancer of Breast [tiab] OR Cancer of the Breast [tiab] OR Mammary Carcinoma*[tiab] OR Mammary Neoplasm*[tiab] OR Breast Carcinoma*[tiab])
(2) (“Mammography" [Mesh] OR Mammogra*[tiab] OR Digital Mammogra*[tiab])
(3) (circumscribed mass*[tiab] OR well circumscribed mass*[tiab] OR well-circumscribed mass*[tiab] OR well defined mass*[tiab] OR well-defined mass*[tiab])
